# Assessing changes in the food retail environment during the COVID-19 pandemic: opportunities, challenges, and lessons learned

**DOI:** 10.1186/s12889-022-12890-x

**Published:** 2022-04-18

**Authors:** Rienna G. Russo, Shahmir H. Ali, Tamar Adjoian Mezzacca, Ashley Radee, Stella Chong, Julie Kranick, Felice Tsui, Victoria Foster, Simona C. Kwon, Stella S. Yi

**Affiliations:** 1 Department of Population Health, NYU Grossman School of Medicine, New York, USA; 2 Department of Social and Behavioral Sciences, School of Global Public Health, NYU, New York, USA; 3 New York, USA; 4 NYU Grossman School of Medicine, New York, USA; 5 Columbia Mailman School of Public Health, New York, USA

**Keywords:** COVID-19, Urban health, Food retail environment, Methodology, Food security

## Abstract

**Background:**

COVID-19 mitigation strategies have had an untold effect on food retail stores and restaurants. Early evidence from New York City (NYC) indicated that these strategies, among decreased travel from China and increased fears of viral transmission and xenophobia, were leading to mass closures of businesses in Manhattan’s Chinatown. The constantly evolving COVID −19 crisis has caused research design and methodology to fundamentally shift, requiring adaptable strategies to address emerging and existing public health problems such as food security that may result from closures of food outlets.

**Objective:**

We describe innovative approaches used to evaluate changes to the food retail environment amidst the constraints of the pandemic in an urban center heavily burdened by COVID-19. Included are challenges faced, lessons learned and future opportunities.

**Methods:**

First, we identified six diverse neighborhoods in NYC: two lower-resourced, two higher-resourced, and two Chinese ethnic enclaves. We then developed a census of food outlets in these six neighborhoods using state and local licensing databases. To ascertain the status (open vs. closed) of outlets pre-pandemic, we employed a manual web-scraping technique. We used a similar method to determine the status of outlets during the pandemic. Two independent online sources were required to confirm the status of outlets. If two sources could not confirm the status, we conducted phone call checks and/or in-person visits.

**Results:**

The final baseline database included 2585 food outlets across six neighborhoods.

Ascertaining the status of food outlets was more difficult in lower-resourced neighborhoods and Chinese ethnic enclaves compared to higher-resourced areas. Higher-resourced neighborhoods required fewer phone call and in-person checks for both restaurants and food retailers than other neighborhoods.

**Conclusions:**

Our multi-step data collection approach maximized safety and efficiency while minimizing cost and resources. Challenges in remote data collection varied by neighborhood and may reflect the different resources or social capital of the communities; understanding neighborhood-specific constraints prior to data collection may streamline the process.

**Supplementary Information:**

The online version contains supplementary material available at 10.1186/s12889-022-12890-x.

## Background

Community mitigation strategies developed to reduce COVID-19 transmission, including social distancing and stay-at-home orders, have uniquely affected food systems and the food retail environment in the United States (US). The food retail environment plays an important role in individual and community health, with substantial associations between food access (i.e., quality, availability and affordability of food) and both prevalence of diet-related diseases and food security [1].

New York City (NYC) was an early epicenter of the pandemic, with 5% of global cases and only 0.1% of the world’s population [2]. One in four NYC residents experienced food insecurity pre-pandemic [3], and research has suggested that the COVID-19 pandemic has exacerbated food access barriers [4]. Evidence emerged indicating that compounding factors, including the closure of the financial district, migration out of Manhattan, decreased travel from China as well as increased fears of viral transmission and xenophobia, may be leading to disproportionate closures of Manhattan’s Chinatown restaurants and small businesses [5, 6]. However, methods to quantify the impact of the pandemic on local food retail environments and strategies to enhance the reliability of such assessments have not yet been documented. Closures of food outlets may have lasting implications for food security, dietary behaviors, and health outcomes [7]. Thus, we developed the COVID-19 Closures (CoClo) project to investigate changes to the operational status (e.g., open, limited service, temporarily closed) of food retailers across different neighborhoods during the pandemic.

While prior research has relied on health/licensing databases to establish a census of food outlets in a geographic region and in-person fieldwork to document the existence and qualitative aspects of stores and restaurants, the application of these methods to assess the physical environment in rapidly evolving conditions and constraints of the pandemic were challenging [8, 9]. Resource (i.e., time, money, and staffing) constraints and fieldwork restrictions (i.e., social distancing, work from home guidelines) limited our ability to rely on in-person assessments and licensing databases alone [10]. Thus, we supplemented existing food assessment methods (i.e., in-person field work, online databases) with innovative strategies (i.e., Google Street View (GSV), web-scraping and phone-based checks, conducted in three languages) [11].

GSV images (i.e., photos of the built environment captured using cars with 360-degree cameras) and web scraping (i.e., extracting data from websites) have also been previously utilized in food retail environment research [12–17]. Though, to our knowledge, the CoClo study is the first of its kind to combine these four methods – health databases, web-scraping, phone-call checks and in-person fieldwork – to minimize their weaknesses as individual strategies and maximize their utility in exploring longitudinal changes to the food environment [18]. While these methods were tested during the pandemic there are opportunities to adopt these methods after the pandemic ends, especially in cases when resources are limited.

Here, we document how our study adapted to the constraints of the pandemic to assess the food retail environment in NYC as an urban center heavily burdened by COVID-19. We outline how we established a baseline universe of food outlets open prior to the pandemic using existing licensing databases. We then detail strategies to safely and efficiently update data on the operational status of the food outlets using a combination of web-scraping, phone-call checks and in-person visits. Lastly, we discuss the challenges we faced in collecting data across diverse neighborhoods, lessons learned for future efforts and opportunities available to utilize data for research or advocacy.

## Methods

### Food outlet sample identification – selecting relevant databases

Consistent with previous studies that utilized health licensing and permits databases to enumerate restaurants and food retailers within a specified location [9, 19], we identified publicly available datasets from local and state public health agencies to enumerate the baseline sample of restaurants and food retailers in NYC. For restaurants, we used the NYC Department of Health and Mental Hygiene (DOHMH) Restaurant Inspections database [20]. The dataset contains names, locations, and inspection results for restaurants, cafeterias, and similar food outlets inspected by the DOHMH up to three years prior to the most recent inspection. We excluded duplicate entries. Though inspection results are usually updated daily, due to the COVID-19 pandemic, the DOHMH suspended restaurant inspections on March 16, 2020 [21]. Therefore, this list was the most comprehensive and up to date list of restaurants available for the NYC prior to the COVID-19 pandemic.

For food retailers, we used the New York State (NYS) Retail Food Stores list, provided by the NYS Department of Agriculture and Markets. The dataset contains names and addresses of food retailers – including convenience/corner stores (also commonly called “bodegas” in NYC), grocery stores, supermarkets, wholesale superstores, bakeries, seafood markets and more –licensed in NYS in 2019 [22]. Food retailers were sampled from this registry. Both datasets were cleaned manually and using Stata (v.15.0, StataCorp LLC, College Station, TX), to check for duplicate entries and errors in names or addresses.

### Neighborhood selection – defining neighborhood types

Due to the constraints of COVID-19 crisis and the multitude of food outlets in NYC, we selected three types of neighborhoods, across two boroughs, to evaluate and compare how the COVID-19 pandemic has affected the food retail landscape. We first selected Chinese ethnic neighborhoods (Chinatown in Manhattan; Sunset Park in Brooklyn). Reporting from news outlets and community partners suggested that the pandemic was having a disproportionate effect on businesses in Chinatown due to decreased tourism and travel from China as well as general fears of contracting the virus, whereby losses in business was not comparable to the actual rates of COVID-19 infection [5, 6].

We selected one higher- and one lower-resourced comparison neighborhood in both Manhattan and Brooklyn based on objective and subjective measures. Indicators from the NYC Community Health Profiles – an in-depth, detailed data source on the 59 Community Districts (CDs) in NYC, developed and maintained by the DOHMH [23] – included in the consideration were key correlates of food access (supermarket to bodega ratio), demographics (population size, foreign born, limited English proficiency), socioeconomic factors (poverty, unemployment, rent burden, uninsured), health behaviors (physical activity, fruit & vegetable, and sugary drink consumption) and chronic conditions (obesity, diabetes, hypertension). No neighborhoods were unequivocally determined to be highest- or lowest-resourced based on aforementioned characteristics. Consequently, we additionally considered anecdotal evidence from research staff and community partner discussions, most of whom are longtime NYC residents, in addition to these factors. These neighborhoods have historically been used as examples of disparities, where the Upper East Side (UES) is often referenced as one of the overserved neighborhoods, in terms of food and medical access comparable to the needs of the residents, in Manhattan and NYC, overall. NYU Center for the Study of Asian American Health has established community partnerships through community health workers, volunteers, and member at organizations including but not limited to the Apicha Community Health Center, Charles B Wang Community Health Center, and the Chinese-American Planning Council [24]. A full list of partnerships can be found here: https://med.nyu.edu/departments-institutes/population-health/divisions-sections-centers/health-behavior/section-health-equity/community-engagement-education/partnerships-coalitions. The final selections of higher- and lower-resourced neighborhoods were the UES and East Harlem in Manhattan, and Park Slope and Brownsville in Brooklyn, respectively (Table 1).

**Table 1:** Characteristics of Comparison Neighborhoods, CoClo Project 2020

Neighborhood	Manhattan			Brooklyn		
	Lower East Side and Chinatown	Upper East Side	East Harlem	Park Slope and Carroll Gardens	Sunset Park	Brownsville
Bodega: Supermarket Ratio^a^	18	5	17	12	45	15
Population	171,103	225,914	124,323	109,351	132,721	84,525
Born outside US	34%	23%	24%	17%	48%	30%
Limited English proficiency	28%	6%	19%	7%	49%	10%
Poverty	18%	7%	23%	10%	29%	28%
Unemployment	8%	4%	11%	6%	8%	14%
Rent Burden	48%	41%	48%	37%	57%	57%
No Health Insurance	11%	4%	12%	4%	22%	12%
Physical activity in past 30 days	77%	87%	68%	86%	68%	74%
At least 1 fruit & vegetable serving/day	88%	94%	84%	94%	87%	80%
≥1 sugary drink/day	16%	13%	29%	14%	24%	35%
Obesity	10%	11%	28%	15%	24%	41%
Diabetes	11%	4%	17%	6%	11%	13%
Hypertension	22%	15%	34%	22%	27%	33%
COVID-19 cases per 100,000^b^	1638	1334	2476	1199	2002	2277

^a^The number of bodegas relative to supermarkets. Calculated from the inverse of the supermarket to bodega ratio, which is what is reported in the NYC Neighborhood Health Atlas

^b^All metrics are at the community district level aside from COVID-19 cases, which are average COVID-19 cases across zip codes included in community districts

### Food outlet sample selection - mapping with Neighborhood Tabulation Areas (NTAs)

**Fig. 1:**
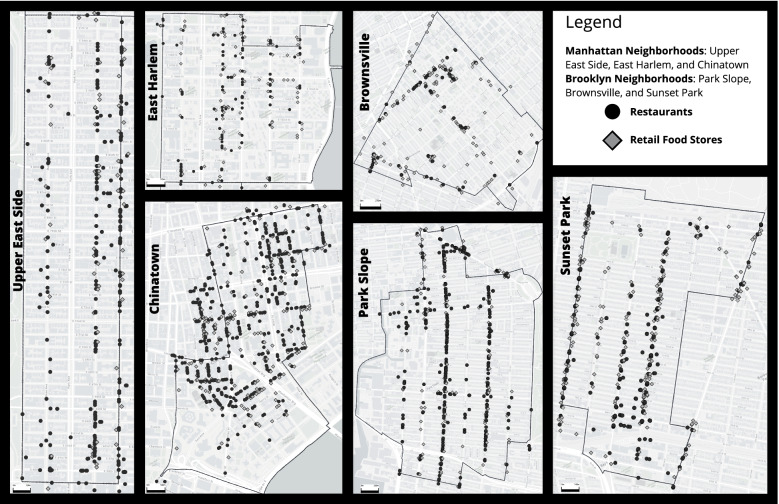
Baseline database of restaurants and retail food stores in six New York City neighborhoods

Restaurant and food retailer databases included geographic information in the form of street addresses and latitude and longitude coordinates. We created maps defining NYC neighborhoods using neighborhood tabulation areas (NTAs) – geographic units created by the DOHMH – on top of which, coordinates of food outlets were plotted. NTAs were purposefully selected to define neighborhoods instead of CDs, referenced previously, for logistical and feasibility reasons. NTAs are smaller geographic regions than CDs, so they were easier to cover given the timeframe of COVID-19 and number of team members available. Because NTA boundaries were created for data purposes, they may not definitively represent neighborhoods. Given these constraints, we extended the NTA boundaries 50 m (m) in an attempt to capture neighborhoods more completely. Additionally, in Chinatown and Sunset Park, the two Chinese ethnic neighborhoods, we expanded boundaries further based on conversations with community partners. In Chinatown, the western NTA-defined border was Bowery Street and Canal Street. This was extended to Mulberry Street and Kenmare Street. In Sunset Park, the south-western NTA-defined border was 60th Street and 6th Avenue. This was extended to 50th Street and 5th Avenue. (Fig. 1) In Chinatown Manhattan, prior research had established the boundaries of the neighborhood during an in-person walk through [25]. However, due to limitations in resources and time-constraints, we did not physically check the boundaries of these neighborhoods, i.e., that Chinese stores were significantly lower across certain streets, prior to initiating data collection.

Using ArcGIS software (ArcGIS [GIS software]. Version 10.0. Redlands, CA), food retailers and restaurants were plotted overlaying the NTA boundaries, which were imported using a DOHMH provided shapefile. https://data.cityofnewyork.us/City-Government/Neighborhood-Tabulation-Areas-NTA-/cpf4-rkhq We first re-drew and extended the boundaries of the NTAs in ArcGIS. Next, we exported all restaurants and food retailers located within the new boundaries to create a census of restaurants and food retailers in the six neighborhoods. Data included in the new lists were the names of the restaurant or food retailers, locations, and phone numbers when available.

### Creating a baseline database – inclusion and exclusion criteria

To develop the baseline lists of restaurants and food retailers open in 2019, we searched for evidence online that the food outlet was open prior to 2020. For the purposes of this study, we defined the pre-pandemic period as prior to January 2020. Though NYS On Pause, the statewide mandated closure of non-essential businesses, did not take effect until March 22, socioeconomic ramifications of COVID-19 began to take effect in Chinatown as early as January 2020 [5, 6]. Research staff searched for evidence that the food outlet was open in 2019, starting with GSV imagery, which contained images from fall of 2019. Additional methods were used if the status could not be ascertained using GSV because something was obstructing the view of the outlet or there were no recent images. Staff then searched for other available evidence with a calendar date in the form of reliable customer reviews, website information, online photos, or reliable news articles. Dates of evidence were recorded, and food outlets were classified as open pre-COVID-19 if they were open prior to 2020. If food outlets were found to be closed in 2019, they were marked as closed pre-COVID-19. Food outlets were marked as ‘not found’ if there was evidence that the food outlet had changed names or ownership in 2019. Stores marked ‘pre-COVID-19 closure’ and ‘not found’ were later excluded from the baseline database. Food outlets confirmed to be open during 2019 were included in the baseline database.

### Data collection – ascertaining the status of food outlets

We created standardized restaurant and food retailer Excel spreadsheets to collect data on the current status of food outlets in the baseline database. Included in the retail food data collection spreadsheet was the tradename (doing business as, “DBA”), location (including the address and coordinates of the business), phone number, status of the store during the COVID-19 pandemic, pre-COVID-19 pandemic status of the store, and extra checks (phone call and in-person) (see Supplemental File). These columns were also included in the restaurant data collection spreadsheet in addition to columns to account for whether the store offered curbside-pickup, contactless delivery, take-out, or dine-in. Both restaurant and food retailer spreadsheets also included a notes column to allow research staff to comment on additional information that did not fit into one of the aforementioned columns.

We used three-step verification system to ascertain the status of all food outlets in this study. The first step was the Google Maps search and online verification. We employed manual web-scraping to identify two independent sources certifying the status of the outlet. If two sources could not confirm the status of the outlet, the next steps involved phone calls and/or in-person visits. Study team members entered data collected on restaurants and food retailers from these sources into the corresponding columns in the Excel spreadsheets. The timeline of the process is available in the Supplemental File. Each step is described in detail below.

To assess the status of food outlets during COVID-19, research staff first searched for the food outlet on Google Maps (https://www.google.com/maps) using the DBA name and address. Any information provided by Google Maps, such as the hours and days the store was open, live visit data (updated real-time information representing how active a location is compared to usual activity managed by Google) [26]; or a temporarily or permanently closed notice was extracted. For some restaurants, Google Maps also provided information about dine-in, take-out and delivery. This data was extracted when available. If Google Maps did not have information available on whether the food outlet was open, (e.g., listed the phone number but no hours; or the store was not recognized) then a note was made that no information was found. For all Google Maps checks, the date the check was conducted was also noted. As Google Maps has a feature enabling users to take notes, we were able to provide real time updates whether a food outlet was open or closed. An additional internet verification step was then conducted to confirm that the Google Maps data was accurate, through a manual data scrape of other online sources, including Google reviews, Yelp, Facebook, Seamless, Grub Hub, the food outlet’s website as well as other websites (e.g., blogs, newspapers). A food outlet was considered open during COVID-19 if there were recent posts or reviews (in the past four months: March – June 2020) about the food outlet. The date of the secondary verification confirming the food outlet as open or closed was also noted. Food outlets were considered ‘temporarily closed’ if they were marked as such on Google or the food outlets’ website. Food outlets were considered ‘indefinitely closed’ if Google had a red ‘permanently closed’ notice or Yelp had an alert that ‘Yelp users have reported this location as closed.’ Food outlets with no evidence online about their current operation status were identified as needing a phone call verification.

Phone numbers were provided in the original restaurant dataset and retrieved from online sources in the food retailer dataset. When calling, research staff verified the name of the food outlet and asked whether they were currently open. If possible, research staff ascertained if the food outlet had closed in the past 6 months and if restaurants were offering delivery, take-out or dine-in services. In the case of no answer, staff noted the time of the call (e.g. morning or afternoon) and called back at a different day and/or time – i.e., if the call was made in the morning, the follow-up call was made in the afternoon. This was especially important for restaurants, which may be open during lunch and evening hours but not in the morning and mid-afternoon. If two calls went unanswered, the food outlet was marked as needing an in-person check. In-person checks were also required for food outlets that had no working phone number (i.e., no longer in service, wrong number, or not available). Some food outlets needed in-language follow-up, particularly those in the ethnic or more racially/ethnically diverse neighborhoods (Chinatown, Sunset Park, East Harlem). Two research staff fluent in Mandarin Chinese and one research staff with conversational Spanish ability conducted these additional calls.

Location data about food outlets needing an in-person check were uploaded to Google Maps using a Google login that was shared with the CoClo team. We utilized the Lists and Notes functions of Google Maps, which allowed individuals in the field wrote notes directly in Google Maps. These notes synched in real-time enabling researchers to split up in the field and keep updated on which outlets had been checked and which remained. We also provided the option of updating data on paper lists, which individuals later uploaded into Google Maps. The in-person check required marking whether food outlets were open, closed, or no longer the same business on the date of the check. If businesses appeared closed at the time of the in-person check, researchers observed the surrounding area and checked whether the hours of operation were posted on the business to judge the status. For example, businesses that appeared closed but had hours available indicating they were open later or on a different day were marked as open. Businesses that were closed with a for sale or rent sign in the window were marked as indefinitely closed while those that appeared vacant but no evidence of permanent closure (e.g., for rent sign) were marked as temporary closed. Some stores required an in-language follow-up when the business sign was only available in Chinese. Photos were taken of these stores and reviewed by research staff with ability to read written Chinese.

Based on the three-step verification process, restaurants and food retailers were classified as ‘Open,’ ‘Temporarily Closed’ or ‘Indefinitely Closed.’ The first round of data collection was completed summer of 2020. We conducted another round of data collection during spring of 2021 to evaluate longitudinal changes. Person-hours, in terms of time spent on each task, were not measured, and depended on the size of the neighborhood and number of food outlets. The online checks and in-person visits tended to take the longest, as all outlets were checked online initially, and some neighborhoods spanned a larger geographic area. The second data collection round was finished faster than the first, suggesting that additional training and experience could improve efficiency.

### Categorizing types of food outlets

Building on prior work, the North American Industry Classification System (NAICS) was adapted to classify food outlets [27]. The NAICS is the standard used to classify business establishments by type of economic activity [28]. Rather than assigning businesses the NAICS codes, the definitions of the NAICS codes were used to guide categorizations of food retailers and restaurants, with some definitions aggregated into a single category (e.g., specialized food store). Definitions of categories used in this project can be found in the Supplemental File.

For restaurants, two new variables were created corresponding to the category of service - limited service (NAICS 722513); full service (NAICS 722511); cafeterias, grills buffets, and buffets (NAICS 722514); snack and nonalcoholic beverage bars (NAICS 722515); and drinking places (NAICS 722410) – and service types – e.g., chain vendors, takeout, casual, fine dining, bakeries, bars. Full service restaurants were then further categorized into 1) Casual-Dining or 2) Fine-Dining, based on definitions outlined by Hwong & Ok (2013) [29].

Stores on the food retailer database were classified into the following groups: liquor stores (NAICS code 445310), smoke shops (NAICS 453991), convenience stores (NAICS 447110 and 445,120), grocery stores (NAICS 445110), specialized food stores (NAICS 445200, 445,210, 445,230, 445,292) and pharmacies (NAICS 446110). Categorization of food retailers was done using the DBA name. Well-known (e.g., Rite Aid, 7-Eleven, Food Stop) and straightforward DBA names (e.g., Fortune Meat Market, Stanley’s Pharmacy, Dubai Smoke Shop) were used to classify food retailers. After this was completed, an online search was used to confirm the type of business for the remaining stores with ambiguous names. Another variable was created to represent whether the food retailer was part of a larger chain. Names were scanned to identify easily recognizable chain food outlets (e.g., Whole Foods Market, Gristedes). Those that could not be identified by name alone were searched online to determine whether they met the DOHMH’s definition of a chain outlet (i.e., 15 locations or more nationally) [30].

#### Finalizing food outlet databases

The food retailers list was cross-checked with the restaurants list to ensure there were no duplicates, and none were found. Businesses that were not primarily food outlets (i.e., bars, hotels, movie theaters, liquor stores, and smoke shops) were excluded. Pharmacies and drug stores such as Duane Reade, CVS, and Rite Aid were included; over the past few years, grocery items have become increasingly available at these locations [31–34]. Moreover, these locations also accept Supplemental Nutrition Assistance Program (SNAP) benefits for food items sold and may be used by low resourced populations as primary food retailers.

Food retailers were linked to the Food and Nutrition Service (FNS) of the United States Department of Agriculture (USDA) SNAP Retailer Locator Database. The database includes retailer names, full addresses and location coordinates. https://usda-fns.hub.arcgis.com/datasets/USDA-FNS::snap-store-locations?geometry=-48.912%2C-9.798%2C19.643%2C74.211 The CoClo database was linked with SNAP Retailer Locator database using retailer name and location data to classify food retailers as either SNAP-authorized retailers or not.

### Analyzing differences in data collection steps by neighborhood

To quantify differences in the data collection steps required across neighborhood, we compared percentages of stores needing call checks, and in-person checks using chi-squared and post-hoc Bonferroni analyses in Stata (v.15.0, StataCorp LLC, College Station, TX). Maps of food outlet closures were created using ArcGIS.

## Results

A summary of the key findings (i.e., closures by neighborhood) has been described elsewhere [35]. In this paper, we present data relating to the Summer 2020 data collection phase in three Manhattan neighborhoods - UES, East Harlem, and Chinatown – and three Brooklyn neighborhoods – Park Slope, Brownsville, and Sunset Park. Across these six neighborhoods, a total of 2218 restaurants and 982 food retailers were initially identified. There were 386 restaurants and 229 food retailers that were excluded because they were either closed or ‘not found’ pre-COVID-19 pandemic. The final baseline database contained 1832 restaurants and 753 food retailers that were open pre-COVID-19 pandemic. There were 1055 restaurants in Manhattan (131 in East Harlem, 285 in the UES, and 639 in Chinatown) and 777 restaurants in Brooklyn (71 in Brownsville, 399 in Park Slope and 307 in Sunset Park), and 329 food retailers in Manhattan (89 in East Harlem, 61 in the UES and 179 in Chinatown) and 424 food retailers in Brooklyn (133 in Brownsville, 109 in Park Slope and 182 in Sunset Park). (Fig. 1) The two lower resourced areas, Brownsville (*N* = 204) and East Harlem (*N* = 220), had the least number of total food outlets.

### Differences in number of steps required for verification of status

We found that ascertaining the status of food outlets was more difficult in certain neighborhoods compared to others. Of the 1832 restaurants across Manhattan and Brooklyn, 78% were verified using the online data extraction process only. There were 404 restaurants (22% of total) that required a phone-call follow-up and 169 (9% of total) that required an in-person follow-up. Only one restaurant required an in-person check without a prior phone call, as all other phone numbers were available from the initial restaurants database. Aggregate neighborhood data is available in Table 2. The UES required the lowest percentage of call checks (12%) and in-person checks (4%), while Sunset Park required the greatest percentage of call checks (50%) and in-person checks (17%). (Supplemental Table 1).

**Table 2:** Call and in-person checks required for food outlets in 6 NYC neighborhoods

	Total		High-resourced Neighborhoods		Low-resourced Neighborhoods		Chinese Ethnic Neighborhoods		*p*-value
	N	%	N	%	N	%	N	%	
Food retailers									
**Call Check**									< 0.001
No	417	55%	138	81%	113	51%	166	46%	
Yes	336	45%	32	19%	109	49%	195	54%	
**In-person Check**									< 0.001
No	532	71%	155	91%	130	59%	247	68%	
Yes	221	29%	15	9%	92	41%	114	32%	
Restaurants									
**Call Check**									< 0.001
No	1428	78%	612	89%	140	69%	676	71%	
Yes	404	22%	72	11%	62	31%	270	29%	
**In-person Check**									< 0.001
No	1663	91%	647	95%	179	89%	837	88%	
Yes	169	9%	37	5%	23	11%	109	12%	

Of the total 753 food retailers across all neighborhoods, 417 stores (55% of total) were completed using the online data extraction process only. A greater percentage of Brooklyn stores required follow-ups (50%) than Manhattan stores (38%). Overall, 218 stores (29% of total) required a call check. There were 221 stores (29% of total) – 103 stores identified during call checks and 118 stores that had no phone number available – that required an in-person follow-up. The UES required the lowest percentage of call checks (15%) and in-person checks (3%). Sunset Park required the greatest percentage of call checks (61%) Brownsville required the greatest percentage of in-person checks (47%). (Supplemental Table 1).

In post-hoc analyses, three patterns emerged. First, lower-resourced neighborhoods had significantly fewer call checks when compared to Chinese ethnic enclave neighborhoods among food retailers and restaurants. Second, higher-resourced neighborhoods required significantly fewer call and in-person checks when compared to lower-resourced and ethnic enclave neighborhoods among food retailers and restaurants. Lastly, for restaurants only, Sunset Park required significantly more call and in-person checks compared to Manhattan Chinatown.

## Discussion

The objective of this paper was to describe how our team adapted to the COVID-19 pandemic mitigation strategies to evaluate the food retail environment in a city that experienced a significant burden of COVID-19 cases, describe challenges in collecting data, lessons learned for future efforts, and opportunities to utilize CoClo data. Web-scraping and GSV images were used to collect information about the operational status of food outlets pre-pandemic and multiple time-points during the pandemic. Phone calls and in-person checks were conducted on a subset of food outlets for which online information was unavailable. Ascertaining the operational status of food outlets was more difficult in certain neighborhoods compared to others. This may reflect the different resources or social capital of the communities. We found that the higher resourced neighborhoods (UES and Park Slope) required fewer phone call and in-person checks for both restaurants and food retailers than other neighborhoods.

Neighborhood selections and definitions in this study were not assessed based on objective measures alone. Because measuring changes for all NYC neighborhoods is resource intensive and difficult to accomplish due to the COVID-19 restraints, three distinct NYC neighborhood types were selected to understand the diversity of experiences. Insight from community partners was leveraged along with data on socioeconomic and health metrics to select six neighborhoods representing higher resourced, lower resourced and Chinese ethnic neighborhoods within NYC. For neighborhood definitions, boundaries defined by the DOHMH were broadened based on anecdotal evidence from research staff and community partners to better delineate meaningful neighborhood boundaries reflective of their social, cultural, and political relevance. This was done to mitigate concerns that geographic borders may not always align with sociocultural boundaries; prior literature has noted that there is no one ideal way to define a neighborhood [36].

We developed a novel three-step verification process, re-imagining and combining existing food environment assessment methods, to achieve an accurate representation of the food environment at multiple timepoints during the COVID-19 pandemic. In-person assessments of all food outlets would have been resource-intensive and difficult due to COVID-19 fears and constraints. Sole reliance on Google and online sources, which may not have the most updated or complete information, would not have provided a complete picture. Consequently, web-scraping and phone call checks were paired with fieldwork allowing for an initial remote assessment important to limiting the number/duration of in-person visits.

For the purposes of this project, January 2020 was used to define the start of COVID-19 epidemic in NYC. Concerns began rising over the threat of the virus in January, leading to decreased business and growing xenophobia in Chinatown prior to the first case reported on March 1st and the NYS On Pause mandate enacted March 22nd. In addition, fears of property damage on the part of small business owners during the early Black Lives Matter protests sparked by George Floyd’s death may have compromised business operations. Researchers evaluating food environments in other regions should consider timing of the virus outbreak, mitigation strategies and social unrest when selecting cut-off dates for inclusion and collection of data.

### Lessons learned

Important lessons were learned when developing this protocol. We found that the harmonization of in-person checks data within the Google Maps platform, through the Lists and Notes functions, allowed researchers to make independent field visits while keeping up to date on which food outlets had been checked by other team members and which were remaining. An advantage of the recent scale-up in delivery and takeout from restaurants was that some secondary verifications could be completed using third-party delivery services – e.g., UberEats, Grub Hub, Seamless and DoorDash. Additionally, when completing phone call checks, we found that some calls needed to be done in-language, i.e., the native language of the outlet owner or employee. When assessing neighborhood food environments, research teams should understand the language requirements within communities and ensure that they have staff or community partners willing and able to conduct checks in-language if needed. Lastly, during our second round of data collection, we found that the online checks were completed more efficiently, as research team members were more comfortable with the extraction process, suggesting that training could reduce the time required to conduct data collection.

### Opportunities for CoClo data and future work

Recent anecdotal evidence has revealed that food retailers have had to adapt to changing circumstances by scaling up their online presence and introducing curbside pickup or contactless delivery options [37]. The resulting shifts in the food retail environment (e.g., increased online grocery shopping) and food purchasing patterns (e.g., decreased intake of food away from home) have major implications for food security, dietary behavior, and health outcomes [38, 39]. The purpose of the CoClo study was to create longitudinal data to describe changes to the food retail environment during the COVID-19 pandemic. Data from this project can be used in future systems science efforts to simulate the effect of food outlet closures on health behaviors and outcomes. Techniques such as network analysis, systems dynamics and agent-based modeling will allow us to understand the complex relationships between environmental factors (e.g., food outlet status), food security, health behaviors, risks and outcomes in the era of the COVID-19 pandemic and post-pandemic periods.

In addition to health concerns related to changes in food retail environment, the economic ramifications among smaller, local restaurants and food retailers as well as the cascading effects on the food system have been largely unexplored. Smaller restaurants and food retailers may lack the financial infrastructure to endure lengthy closures or scale-up take-out options compared to chain stores and established franchises. These small businesses are a vital part of the US food system; in April of 2020, the New York Times reviewed of how initial closures left farmers with no buyers, resulting in staggering amounts of food waste [40]. The Paycheck Protection Program (PPP) of Coronavirus Aid, Relief and Economic Security (CARES) Act was designed to help struggling small businesses during the COVID-19 pandemic-related government mandated closures. Though businesses with 500 employees or more were ineligible to receive funds, loopholes in the program guidelines led to larger food corporations receiving over $10 million in loans [41]. Without assistance, these small food retailers may permanently shut their doors. The absence of these retailers from the US food system has unknown but potentially devastating effects. Data from CoClo may be used in future advocacy work for government relief programs and other economic assistance.

Given the resource constraints of the pandemic, we were unable to evaluate further business aspects, i.e., online sales and changes in food item prices, or test the validity of this three-step data collection process. Novel methods to assess patronage include mobile phone data that can enable researchers to identify the frequency of visits to locations. This data has been important during the pandemic for contract tracing and monitoring migration patterns. We are unaware of current research efforts to use mobile phone data to evaluate changes in patronage of food businesses, but this presents an opportunity that might be explored in future work. Furthermore, future data collection efforts of business closures using the three-step process should validate the method against in-person only checks. Ground-truthing or on-site verification of food outlets has been previously established as the gold standard for food retail environment surveillance [42–44]. Another opportunity could be to re-order the phone call and internet checks to test how robust the original method was. A validation study could support the use of this method in other settings and contexts.

### Limitations

Despite our efforts to adapt to the circumstances, we report some shortcomings to our methods. There may be instances where stores were incorrectly categorized as indefinitely closed when they were temporarily closed during COVID-19 mandates. However, temporary closures may lead to lasting closures, as seen already in some cities where restaurants closed during the stay-at-home order were forced to close permanently [45, 46]. Moreover, there may be misclassification whereby businesses were marked as temporarily closed during in-person checks but were open and operating under extremely limited hours or on a takeout basis only. In future work, if these methods were applied to different settings in which operations were not able to be checked, then an ‘uncertain’ category may be applicable. Because the objective of this project was to determine changes in the food retail environment during the COVID-19 pandemic, we felt that accounting for any closure was sufficient, especially as the initial documentation was done when restaurants and stores were beginning to reopen. Additionally, we are hesitant to attribute all of the changes directly to COVID-19 mandates. Some closures may be due to construction. Moreover, Black Lives Matter protests, which started May 2020 and continued through August 2020, may have contributed to some changes. Food retailers that opened since June 2019 were not accounted for, as this was the last date the food retailer database was updated at the time of data extraction and analysis. It was not known when the database would be updated, and given the pressing nature of this project, the 2019 data were used. In some instances, food outlets underwent a name change, which made it more challenging to determine the status, i.e. if the food outlet had closed or remained open under a different name.

## Conclusions

The constantly evolving COVID-19 crisis has caused research design and methodology to fundamentally shift, requiring researchers to develop creative, flexible, adaptable strategies to address emerging and existing public health problems. Construction of the CoClo dataset highlights our innovate efforts to combine existing and emerging food assessment methods to conduct research safely and efficiently during the pandemic. These methods may be replicated in other locations, specifically cities within the US that are experiencing high burdens of COVID-19 cases, to understand changes to the food retail environment. Data may also be used by community and governmental partners to advocate for additional funding to community programs around food insecurity or to bolster support for small businesses. Moreover, such methods may enable local stakeholders to better understand the economic ramifications of COVID-19 in their community. Future research could integrate data from this study into simulation models to project how changes to the food retail environment impact dietary behaviors of neighborhood residents, and the US food system.

## Supplementary Information

**Additional file 1.**

## Data Availability

The data supporting the conclusions of this article are available upon request.

## Abbreviations

NYC: New York City
US: United States
CoClo: COVID-19 Closures Project
GSV: Google Street View
DOHMH: Department of Health and Mental Hygiene
NYS: New York State
CD: Community District
UES: Upper East Side
NTA: Neighborhood Tabulation Areas
DBA: Doing Business As
NAICS: North American Industry Classification System
SNAP: Supplemental Nutrition Assistance Program
FNS: Food and Nutrition Service
USDA: United States Department of Agriculture
PPP: Paycheck Protection Program
CARES: Coronavirus Aid, Relief and Economic Security Act

## References

[1] Black C, Moon G, Baird J (2014). Dietary inequalities: what is the evidence for the effect of the neighbourhood food environment?. Health Place.

[2] McKinley J. New York City Region Is Now an Epicenter of the Coronavirus Pandemic. The. New York Times. 2020.

[3] Mann B (2020). In New York City, 2 Million Residents Face Food Insecurity.

[4] Wolfson JA, Leung CW. Food Insecurity and COVID-19: Disparities in Early Effects for US Adults. Nutrients. 2020;12(6). 10.3390/nu12061648 PMC7352694 32498323

[5] Coronavirus SA (2020). a fire and anxiety in the Chinese community.

[6] Barron J. Coronavirus in N.Y.: Without Chinese tourists, business sags. The New York Times. 2020.

[7] Leone LA, Fleischhacker S, Anderson-Steeves B, Harper K, Winkler M, Racine E (2020). Healthy food retail during the COVID-19 pandemic: Challenges and future directions. Int J Environ Res Public Health.

[8] Lytle LM, A. Measures Registry User Guide: Food Environment. Washington (DC): National Collaborative on Childhood. Obes Res. 2017.

[9] Moudon AV, Drewnowski A, Duncan GE, Hurvitz PM, Saelens BE, Scharnhorst E (2013). Characterizing the food environment: pitfalls and future directions. Public Health Nutr.

[10] Barroga E, Matanguihan GJJJoKms. Fundamental Shifts in Research, Ethics and Peer Review in the Era of the COVID-19. Pandemic. 2020;35(45). 10.3346/jkms.2020.35.e395 PMC7683245 33230986

[11] Yi S, Russo R, Liu B, Kum S, Rummo P, Li Y. Characterising urban immigrants' interactions with the food retail environment. Public Health Nutr. 2020. 10.1017/S1368980020002682 PMC8565613 32895069

[12] Rzotkiewicz A, Pearson AL, Dougherty BV, Shortridge A, Wilson N (2018). Systematic review of the use of Google Street View in health research: major themes, strengths, weaknesses and possibilities for future research. Health Place.

[13] Burgoine T, Harrison F (2013). Comparing the accuracy of two secondary food environment data sources in the UK across socio-economic and urban/rural divides. Int J Health Geogr.

[14] Fleischhacker SE, Evenson KR, Sharkey J, Pitts SBJ, Rodriguez DA (2013). Validity of secondary retail food outlet data: a systematic review. Am J Prev Med.

[15] Cohen N, Chrobok M, Caruso O (2020). Google-truthing to assess hot spots of food retail change: A repeat cross-sectional Street View of food environments in the Bronx, New York. Health Place.

[16] Google. Google Street View 2020 [Available from: https://www.google.com/streetview/explore/.

[17] Hillen J. Web scraping for food price research. Br Food J. 2019.

[18] Ali SH, Imbruce VM, Russo RG, Kaplan S, Stevenson K, Mezzacca TA (2021). Evaluating closures of fresh fruit and vegetable vendors during the covid-19 pandemic: methodology and preliminary results using omnidirectional street view imagery. JMIR Formative Res.

[19] Daepp MI, Black J (2017). Assessing the validity of commercial and municipal food environment data sets in Vancouver, Canada. Public Health Nutr.

[20] New York City Department of Health and Mental Hygiene. DOHMH New York City Restaurant Inspection Results 2020 [Available from: https://data.cityofnewyork.us/Health/DOHMH-New-York-City-Restaurant-Inspection-Results/43nn-pn8j.

[21] Silvestri P (2020). Health Department suspends restaurant inspections, and that’s A-OK with a lot of restaurateurs.

[22] Retail Food Stores [Internet]. 2019 [cited May 20, 2020]. Available from: https://data.ny.gov/Economic-Development/Retail-Food-Stores/9a8c-vfzj.

[23] New York City Neighborhood Health Atlas [Available from: https://public.tableau.com/profile/nyc.health#!/vizhome/NewYorkCityNeighborhoodHealthAtlas/NeighborhoodData.

[24] Trinh-Shevrin C, Kwon SC, Park R, Nadkarni SK, Islam NS (2015). Moving the dial to advance population health equity in New York City Asian American populations. Am J Public Health.

[25] Imbruce V (2015). From Farm to Canal Street: Cornell University Press.

[26] Google My Business. Help: Popular times, wait times, and visit duration 2020 [Available from: https://support.google.com/business/answer/6263531?hl=en.

[27] Ohri-Vachaspati P, Martinez D, Yedidia MJ, Petlick N (2011). Improving data accuracy of commercial food outlet databases. Am J Health Promot.

[28] United States Office of Management and Budget. North American Industry Classification System 2017 [Available from: https://www.census.gov/eos/www/naics/.

[29] Hwang J, Ok C (2013). The antecedents and consequence of consumer attitudes toward restaurant brands: A comparative study between casual and fine dining restaurants. Int J Hospitality Manage.

[30] NYC Department of Health and Mental Hygiene. De Blasio Administration Announces New Calorie Labeling Rules 2017 [Available from: https://www1.nyc.gov/site/doh/about/press/pr2017/calorie-label-rules.page.

[31] Acosta G. Walgreens Introduces Drive-Thru Grocery Shopping: Progressive Grocer; 2020 [Available from: https://progressivegrocer.com/walgreens-introduces-drive-thru-grocery-shopping.

[32] Japsen B. Walgreens and Kroger To Test Groceries At Drugstores: Forbes; 2018 [Available from: https://www.forbes.com/sites/brucejapsen/2018/10/02/walgreens-and-kroger-to-test-grocery-and-food-pickup-at-drugstores/#238e1d796f3c.

[33] Consumer Reports. Should you buy groceries at the drug store? 2014 [Available from: https://www.consumerreports.org/cro/news/2014/04/should-you-buy-groceries-at-the-drug-store/index.htm.

[34] Canon G. 'Food deserts' become 'food swamps' as drugstores outsell major grocers 2019 [Available from: https://www.theguardian.com/us-news/2019/jun/04/food-swamps-cvs-outsells-trader-joes-whole-foods-processed-shopping.

[35] Yi S, Ali S, Russo R, Foster V, Radee A, Chong S, Tsui F, Kranick J, Lee D, Imbruce V, Mezzacca T (2020). Changes to the Food Retail Environment due to COVID-19: A Snapshot of the New York City Experience, May to July 2020. Under Review.

[36] Flowerdew R, Manley DJ, Sabel CE (2008). Neighbourhood effects on health: does it matter where you draw the boundaries?. Soc Sci Med.

[37] Bogost I. The Supermarket After the Pandemic. The Atlantic. 2020.

[38] Taparia H. How Covid-19 Is Making Millions of Americans Healthier. The. New York Times. 2020.

[39] Redman R. Online grocery sales to grow 40% in 2020. Supermarket News. 2020.

[40] Yaffe-Bellany D, Corkery M. Dumped milk, smashed eggs, plowed vegetables: Food waste of the pandemic. The. New York Times. 2020;11.

[41] Wallace A. Shake Shack, Ruth's Chris and other chain restaurants got big PPP loans when small businesses couldn't: CNN; 2020 Available from: https://www.cnn.com/2020/04/19/business/small-businesses-ppp-loans-chain-restaurants/index.html.

[42] Caspi CE, Friebur R (2016). Modified ground-truthing: an accurate and cost-effective food environment validation method for town and rural areas. Int J Behav Nutr Phys Act.

[43] Wong MS, Peyton JM, Shields TM, Curriero FC, Gudzune KA (2017). Comparing the accuracy of food outlet datasets in an urban environment. Geospat Health.

[44] de Menezes MC, de Matos VP, de Pina MF, de Lima Costa BV, Mendes LL, Pessoa MC (2021). Web Data Mining: Validity of Data from Google Earth for Food Retail Evaluation. J Urban Health.

[45] Kelso A (2020). Coronavirus has already shuttered 16K restaurants, Yelp says: Restaurant Dive.

[46] Rowe J (2020). Yelp economic average uncovers a drop in total business closures and a rise in permanent closures during the second quarter: Yelp.

