# Supplementary material for: Assessing changes in the food retail environment during the COVID-19 pandemic: opportunities, challenges, and lessons learned
Source: BMC Public Health. 2022 Apr 18;22:778. doi: 10.1186/s12889-022-12890-x (PMC9014275; doi:10.1186/s12889-022-12890-x)
Supplement: Supplementary file 1 — Additional file 1. [file 12889_2022_12890_MOESM1_ESM.docx]

**
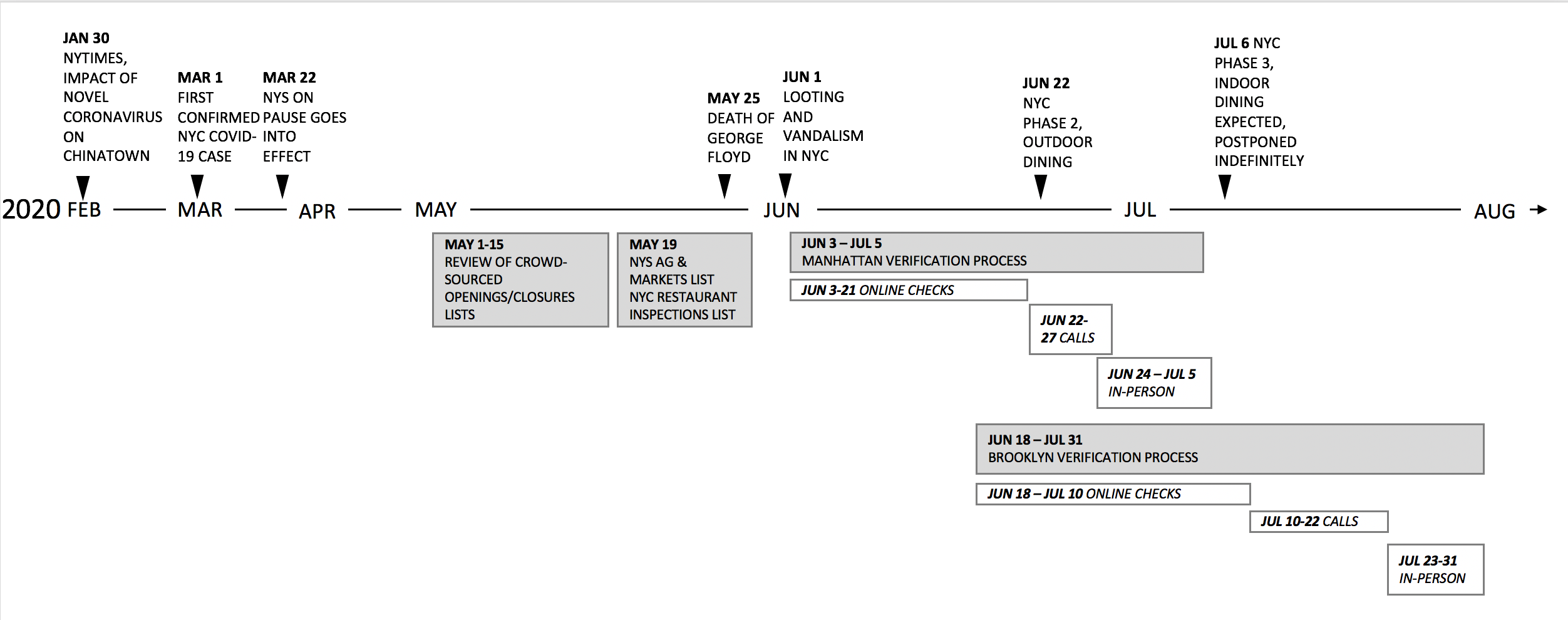
Figure 1.** Timeline of CoClo Project Milestones

**Supplemental Figure 2**. Data extraction spreadsheet categories for restaurants and retail food stores.

1.
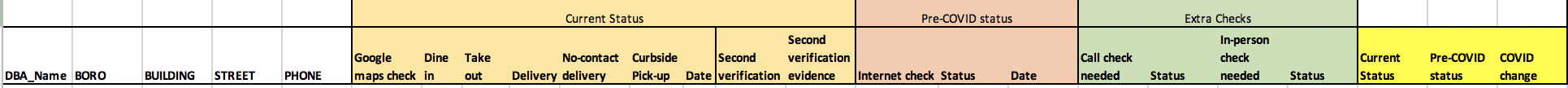
Restaurant extraction categories.
2. Retail food categories.


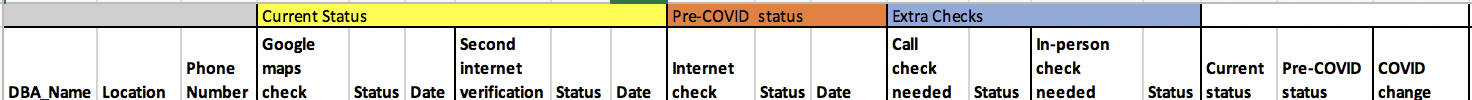


**Supplemental File 3**. Category Definitions of Food Retailers and Restaurants.

*Food retailer definitions:*

- **Liquor store**:
  - Defined by NAICS code 445310: establishments primarily engaged in retailing packaged alcoholic beverages, such as ale, beer, wine, and liquor.
- **Smoke shop**:
  - Defined by NAICS code 453991: establishments primarily engaged in retailing cigarettes, cigars, tobacco, pipes and other smokers’ supplies
    - Includes cigar stores, smokers’ supply stores, cigarette stands (i.e., permanent), and tobacco stores
- **Convenience store**:
  - Defined by NAICS code 445120: establishments known as convenience stores or food marts (except those with fuel pumps) primarily engaged in retailing a limited line of goods that generally includes milk, bread, soda, and snacks.
  - Defined by NAICS code 447110: establishments engaged in retailing automotive fuels (e.g., diesel fuel, gasohol, gasoline) in combination with convenience store or food mart items.
  - Bodegas and delis are included
- **Grocery store**:
  - Defined by NAICS code 445110: establishments generally known as supermarkets and smaller grocery stores primarily engaged in retailing a general line of food, such as canned and frozen foods; fresh fruits and vegetables; and fresh and prepared meats, fish, and poultry. Included in this industry are delicatessen-type establishments primarily engaged in retailing a general line of food
- **Specialized food stores:**
  - Defined by NAICS code 445200: establishments primarily engaged in retailing specialized lines of food, such as retail bakeries, meat and seafood markets, dairy stores, and produce markets
- **Pharmacy**:
  - Defined by NAICS code 446110: establishments engaging in retailing prescription or nonprescription drugs and medicines

*Restaurant definitions:*

- **Limited Service** [722513]
  - Establishments primarily engaged in providing food services (except snack and nonalcoholic beverage bars) where patrons generally order or select items and pay before eating. Food and drink may be consumed on premises, taken out, or delivered to the customer's location. Some establishments in this industry may provide these food services in combination with selling alcoholic beverages.
  - Types
    - **Chain vendor – national**: A restaurant with more than 10 units nationwide. This will usually be pretty straightforward (McDonalds, Chipotle, etc.)
    - **Chain vendor – regional**: A restaurant with more than 10 units in a specific region. For example, a restaurant with chains only in Pennsylvania, NYC, other small areas in the Northeast, etc.
    - **Takeout – Asian**: Any other type of limited service vendor that focuses mainly on Asian foods/cuisines. For now, you can include ALL types of Asian cuisines (including South and Southeast Asian) – we can parse out lately too. You can use the “Cuisine” tab to help you with thus, but also consult other ways of checking.
    - **Other**: Any other type of limited service vendor that focuses mainly on non-Asian/Other cuisines. You can use the “Cuisine” tab to help you with thus, but also consult other ways of checking.
- **Full Service [722511]**
  - Establishments primarily engaged in providing food services to patrons who order and are served while seated (i.e., waiter/waitress service) and pay after eating. These establishments may provide this type of food service to patrons in combination with selling alcoholic beverages, providing carryout services, or presenting live nontheatrical entertainment.
  - Types
    - **Casual Dining:** (Hwang, Ok 2013) “A casual dining restaurant creates a casual atmosphere where patrons enjoy dining-out at moderate prices … a casual dining restaurant serves a casual atmosphere with the above average guest check of $15” – Although we won’t be able to figure out the average guest check, use this broad definition to help guide the distinction
    - **Fine Dining:** (Hwang, Ok 2013) “a fine dining restaurant provides an elegant, even luxurious, atmosphere where customers pay a relatively high price … a fine dining restaurant provides a luxurious atmosphere with the above average guest check of $50” – Although we won’t be able to figure out the average guest check, use this broad definition to help guide the distinction
    - **Other:** Anything else (you probably might not have much or anything in this category if you are able to split all the full service restaurants between casual/fine)
- **Cafeterias, Grill Buffets, and Buffets** [722514]
  - Establishments, known as cafeterias, grill buffets, or buffets, primarily engaged in preparing and serving meals for immediate consumption using cafeteria-style or buffet serving equipment, such as steam tables, refrigerated areas, display grills, and self-service nonalcoholic beverage dispensing equipment. Patrons select from food and drink items on display in a continuous cafeteria line or from buffet stations.
  - Types
    - **Cafeteria:** A cafeteria like vendor that is INSIDE something else (e.g. university cafeteria, prison cafeteria etc.)
    - **Buffet / Other:** Other types of buffet/cafeteria-style stand-alone vendors
- **Snack and Nonalcoholic Beverage Bars** [722515]
  - Establishments primarily engaged in (1) preparing and/or serving a specialty snack, such as ice cream, frozen yogurt, cookies, or popcorn, or (2) serving nonalcoholic beverages, such as coffee, juices, or sodas for consumption on or near the premises. These establishments may carry and sell a combination of snack, nonalcoholic beverage, and other related products (e.g., coffee beans, mugs, coffee makers) but generally promote and sell a unique snack or nonalcoholic beverage.
    - **Desserts/Bakery**: Vendors focused on selling things like ice cream, frozen yogurt, cakes/bakery type stuff, etc., basically desserts and confectionaries
    - **Café**: Vendor that is primarily focused on selling coffee, but might also have some small bakery stuff they’re selling, but the key thing is the coffee.
    - **Juice/Tea**: Juice bar, bubble tea places, etc. – basically vendors focused on selling juice and tea like beverages
    - **Other**: Anything else
- **Drinking Places (Alcoholic Beverages)** [722410]
  - Establishments known as bars, taverns, nightclubs, or drinking places primarily engaged in preparing and serving alcoholic beverages for immediate consumption. These establishments may also provide limited food services.
    - Night Club / Karaoke: If the place seems like a night club type vendor, or if the drinking is pinned down to something else (like a dance hall, karaoke, or general night club etc.)
    - Bar / Other: Any standard bar, or other drinking venue. NOTE: categorize this as Drinking place only if you feel like the food services are relatively limited – if they have a pretty comprehensive food menu, or are a bar + grill, put this as full service / limited service / etc. (food vendor).

| **Supplemental Table 1**. Call and in-person checks required for food outlets in 6 NYC neighborhoods. | | | | | | | | | | | | | | | |
| --- | --- | --- | --- | --- | --- | --- | --- | --- | --- | --- | --- | --- | --- | --- | --- |
|  | Total | | East Harlem | | Upper East Side | | Chinatown | | Sunset Park | | Park Slope | | Brownsville | |  |
|  | N | % | N | % | N | % | N | % | N | % | N | % | N | % | p-value |
| FOOD RETAILERS | |  |  |  |  |  |  |  |  |  |  |  |  |  |  |
| **Call Check** |  |  |  |  |  |  |  |  |  |  |  |  |  |  | < 0.001 |
| No | 417 | 55% | 56 | 63% | 52 | 85% | 95 | 53% | 71 | 39% | 86 | 79% | 57 | 43% |  |
| Yes | 336 | 45% | 33 | 37% | 9 | 6% | 84 | 47% | 111 | 61% | 23 | 22% | 76 | 57% |  |
| **In-person Check** | | | |  |  |  |  |  |  |  |  |  |  |  | < 0.001 |
| No | 532 | 71% | 59 | 66% | 59 | 97% | 122 | 68% | 125 | 69% | 96 | 88% | 71 | 53% |  |
| Yes | 221 | 29% | 30 | 34% | 2 | 3% | 57 | 32% | 57 | 31% | 13 | 12% | 62 | 47% |  |
| RESTAURANTS | | | | | | | | | | | | | | | |
| **Call Check** |  |  |  |  |  |  |  |  |  |  |  |  |  |  | < 0.001 |
| No | 1,428 | 78% | 96 | 73% | 250 | 88% | 521 | 82% | 155 | 50% | 362 | 91% | 44 | 62% |  |
| Yes | 404 | 22% | 35 | 27% | 35 | 12% | 118 | 18% | 152 | 50% | 37 | 9% | 27 | 38% |  |
| **In-person Check** | | | |  |  |  |  |  |  |  |  |  |  |  | < 0.001 |
| No | 1,663 | 91% | 113 | 86% | 275 | 96% | 581 | 91% | 256 | 83% | 372 | 93% | 66 | 93% |  |
| Yes | 169 | 9% | 18 | 14% | 10 | 4% | 58 | 9% | 51 | 17% | 27 | 7% | 5 | 7% |  |
